# Anatomical variations of the pancreatic blood vessels in patients with diabetes/metabolic syndrome

**DOI:** 10.5339/qmj.2025.48

**Published:** 2025-06-09

**Authors:** Anastasiya Spaska, Bogdan Grytsuliak

**Affiliations:** 1 Department of Basic Sciences, Ajman University, Ajman, United Arab Emirates; 2 Department of Anatomy and Physiology, Vasyl Stefanyk Precarpathian National University, Ivano-Frankivsk, Ukraine *Email: anast.spaska@gmail.com

**Keywords:** Pancreas, arteries, endocrinology, public health, Middle East

## Abstract

**Introduction:**

Diabetes is a major public health concern that affects millions of individuals worldwide, and given the increasing prevalence of this disease, there is a critical need to better understand its pathophysiology, which could lead to improved management strategies to mitigate its effects on society. The aim of this study was to investigate the vascular anatomy of the pancreas and classify the arterial variations of pancreatic blood flow, as well as to determine the correlation between these variations and the occurrence of diabetes and metabolic syndrome (MS).

**Methods:**

This study used multidetector computed tomography (MDCT) angiography to assess the vascular anatomy of the pancreas in a total of 100 participants. The variations were classified based on the origin and course of the pancreatic arteries, and the imaging data were recorded and analyzed.

**Results:**

The study identified three major types of arterial variations. The dorsal pancreatic artery (DPA) was observed to arise from the splenic artery (SPA), common hepatic artery (CHA), and superior mesenteric artery (SMA). The prevalence of arterial variations in the observed population (single-center study) in this research was found to be different from that reported in previous studies conducted on other populations. Specifically, the study found a higher incidence of DPA variations arising from the SPA (in 73% of the participants). The origin from the SMA was seen in 24% of patients and from the CHA in 3% of patients. The length, width, and other characteristics of the pancreatic arteries were also carefully documented. The study also found no significant correlation between arterial variations and the presence of diabetes mellitus or MS. One of the variations was found to display minor constriction but was not significant enough to be considered pathological.

**Conclusion:**

The study revealed the utility of MDCT imaging as a reliable tool for studying pancreatic arterial blood flow. This study contributed to the existing body of knowledge about the vascular anatomy of the pancreas and provided valuable insights for future research in this area.

## Introduction

Pancreatic disorders, including pancreatic cancer, pancreatitis, and diabetes, are significant health concerns worldwide. Diabetes mellitus (DM) and metabolic syndrome (MS) are becoming increasingly prevalent worldwide, with a high morbidity and mortality rate, and understanding the associated risk factors is critical for developing effective prevention and treatment strategies.^1^

The rate of increase in the incidence of diabetes can be considered a global problem. According to Lovic et al., the International Diabetes Federation has already recorded 537 million adults in the world living with diabetes in 2022. In 2014, the number of patients was 422 million adults worldwide suffering from diabetes.^2^ The overall prevalence of diabetes among the 20–79 age group is 8.8%. In addition, 1 in 2 people are unaware they have the disease.^3^ Appanraj et al. emphasize that MS is one of the most urgent problems of modern medicine, particularly in the fields of endocrinology, dietetics and nutrition, cardiology, and nephrology.^4^ Basically, MS affects 25%–35% of the population, and among people over 60 years old, it is more than 40%.

The pathophysiology of these diseases is closely related to the vascular anatomy of the pancreas, making a comprehensive understanding of this anatomy crucial for diagnosis and treatment.^1^ The variations in arterial blood supply to the pancreas, being relatively common, are also of great importance for surgical procedures. However, there was limited information available on the prevalence of these variations, particularly within specific ethnic groups. This research addressed this knowledge gap by providing insights into the prevalence of arterial variations of the pancreas among the Eastern European population. This study’s findings potentially have significant implications for the diagnosis and treatment of pancreatic diseases, as well as for surgical planning. Therefore, the article’s topic was highly relevant to current medical practice and research efforts.

According to El Mesmoudi et al.,^3^ a significant proportion of the adult population in the world, especially in the Middle East, has been found to suffer from diabetes, with a prevalence rate of 17.3% in the United Arab Emirates (UAE); however, the sample in this study has not been fully representative of the broader population. A study conducted by Sulaiman et al.^5^ investigated the prevalence of obesity in the UAE population and found that it was alarmingly high. This highlighted the urgent need for effective public health strategies to address this issue and mitigate the risk of developing MS and DM in the population. Mahmoud and Sulaiman^6^ wrote about the need to implement early intervention strategies and routine screening tests to prevent and treat MS in high-risk groups. Multidetector computed tomography (MDCT) can be a useful tool for assessing arterial variations and the vascular anatomy of the pancreas, but it should not be considered a routine screening test for DM or MS. While MDCT is a highly accurate method, its use is limited by factors such as high cost, the need for specialized equipment, and the expertise required from healthcare providers. For early diagnosis of diabetes and MS, simpler and less invasive tests, such as blood glucose measurements and assessments of insulin levels or glycated hemoglobin, are more suitable and accessible for widespread use. Although MDCT can be valuable in specific cases, more affordable and cost-effective methods are necessary for routine screening of these conditions. However, it should be noted that the exact methods of such interventions were not described in detail in the study. Further research is needed to identify effective tests and interventions and develop clear guidelines for their implementation.

Mohamad^7^ described in his work the main risk factors of MS, such as genetics, insulin resistance, abdominal obesity, and glucose intolerance. The vascular supply to the pancreas has been overlooked in this research. Assessing differences in the lifestyle and ethnicity among the UAE population, as mentioned by Jayaraj et al.,^8^ could be used for early detection of DM in the high-risk group, though this way of screening did not provide enough specificity. The individual variations in pancreatic blood flow were studied by Kumar et al.,^9^ who tried to provide a systematic description of the topic. The research, however, was purely anatomical. To date, there has been no comprehensive study that describes the impact of the vascular anatomy of the pancreas on human health and well-being. Based on that, there was a critical need to explore this topic to fully understand its importance.

The goal of the current study was to investigate the correlation between anatomical variations in pancreatic blood flow and the development of diabetes and MS. By identifying the potential relationship between these factors, it was also important to contribute to a better understanding of the pathophysiology of diabetes.

The purpose of this study was to evaluate the prevalence of anatomical variations of pancreatic blood supply (arteries and veins) and their association with diabetes and MS. The authors hypothesized that unexplained carbohydrate-metabolism disorders may be due to variant pancreatic vessel anatomy.

## Materials and Methods

The research was conducted retrospectively based on the guidelines outlined by the radiology department of the Ivano-Frankivsk Oblast Clinical Hospital (Ukraine). This retrospective study used MDCT angiography scans from patients with diabetes and MS to assess pancreatic vessel anatomy.

Altogether, the authors collected and analyzed data obtained from the clinical histories of 100 individuals (dated between 2018 and 2023). Out of them, 50 had been diagnosed with DM and MS (23 males and 27 females), according to the American Diabetes Association criteria for diabetes and the National Cholesterol Education Program’s Adult Treatment Panel III guidelines for MS. The other 50 participants (25 males and 25 females) were controls without DM or MS but had abdominal MDCT for other reasons. Informed consent for the use of their data has been obtained from each participant prior to the study. The authors selected data from the participants aged between 40 and 60 years. Exclusion criteria included a history of acute or chronic pancreatitis, any prior pancreatic surgery, pancreatic tumors or cysts, congenital pancreatic abnormalities, portal hypertension, or cirrhosis, which could potentially affect pancreatic blood flow. The study was conducted in accordance with the principles of the Declaration of Helsinki and was approved by the Bioethics Committee of the Vasyl Stefanyk Precarpathian National University.

Images of the MDCT angiography were obtained with a Philips MX8000 IDT 16 CT Scanner using nonionic iodinated contrast with an automatic injector (CT 9000-ADV Power Injector). The blood flow types were classified based on the observed contrast-enhanced patterns. The types were categorized and determined by two radiologists, blinded to the clinical information of the participants, who reviewed the images independently and reached a consensus. The data obtained have been analyzed using IBM SPSS Statistics version 27.0. Microsoft Office Excel 2019 was also used. Descriptive statistics were used to describe the characteristics of the study population. The chi-squared test was used to compare the prevalence of anatomical variations between the relatively healthy and DM/MS groups. Logistic regression analysis was performed to determine the association between pancreatic blood flow types and the risk of diabetes and MS. The results were considered statistically significant if the *p*-value was less than 0.05.

## Results

The relationship between arterial variations of pancreatic blood flow and the occurrence of diabetes and MS was analyzed. As a result, the authors confirmed that the prevalence of the different arterial configurations did not differ significantly between the group of patients with diabetes and MS and the healthy control group, but the study provided several different valuable findings.

The arterial configurations of the pancreaticoduodenal (PD) complex were identified and categorized based on their anatomical variations. Three different types of PD arterial configurations were found. Type I was the most common (*n* = 73, 73%), followed by Type II (*n* = 24, 24%) and Type III (*n* = 3, 3%). In Type I, the dorsal pancreatic artery (DPA) originated from the splenic artery (SPA). In Type II, the DPA was a branch of the superior mesenteric artery (SMA). In Type III, the DPA originated from the common hepatic artery (CHA), while the inferior PD artery was a branch of the SMA. The three major types of anatomical arterial variations identified in this study were carefully studied and recorded by two experienced radiologists. This subsequently allowed detailed analysis and comparison of the vascular anatomy of the pancreas between the two groups.

Obesity is widely recognized as a significant risk factor for the development of both MS and type 2 diabetes. This chronic condition, characterized by the excessive accumulation of body fat, disrupts the normal function of several bodily systems, particularly the endocrine and metabolic systems. Obesity leads to insulin resistance, where the body becomes less responsive to insulin, resulting in elevated blood glucose levels, which is a primary mechanism for the development of diabetes. Furthermore, excess weight contributes to systemic inflammation and impaired vascular function, both of which are important factors in the development of MS. Since obesity is linked to other conditions such as hypertension, dyslipidemia, and glucose metabolism disorders, it serves as a key factor in the onset of these chronic diseases, which can have serious long-term health consequences.

**Figure 1:**
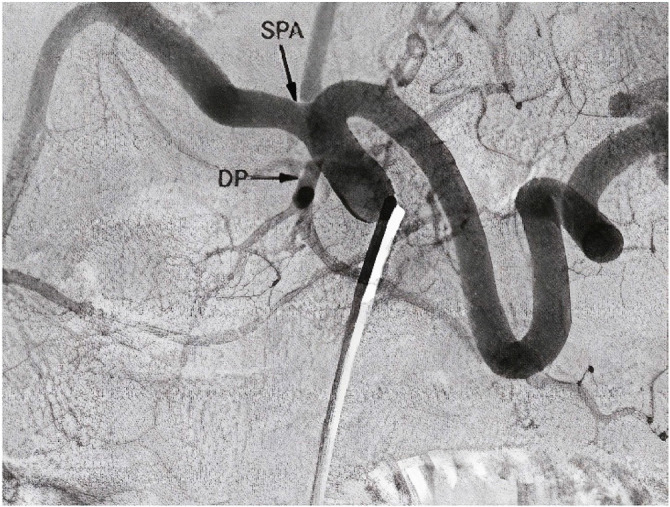
Type I arterial configuration. Note: SPA: splenic artery, DP: dorsal pancreatic artery. Source: Okahara et al.^10^

The following image depicts Type I variation, which was the most common PD arterial configuration found in this study, with a prevalence of 73% (Figure 1). In this configuration, the DPA originated from the SPA, which is the main artery supplying blood to the spleen. The DPA then coursed anteriorly toward the head of the pancreas, where it anastomosed with the PD artery to form the PD arcade. The caliber of the DPA was variable, ranging from 0.7 mm to 2 mm, with an average diameter of 1.2 mm. The SPA and its branches also supplied blood to the stomach and greater omentum. This variation was observed in 33 of the healthy participants and 40 participants from the DM/MS group.

**Figure 2:**
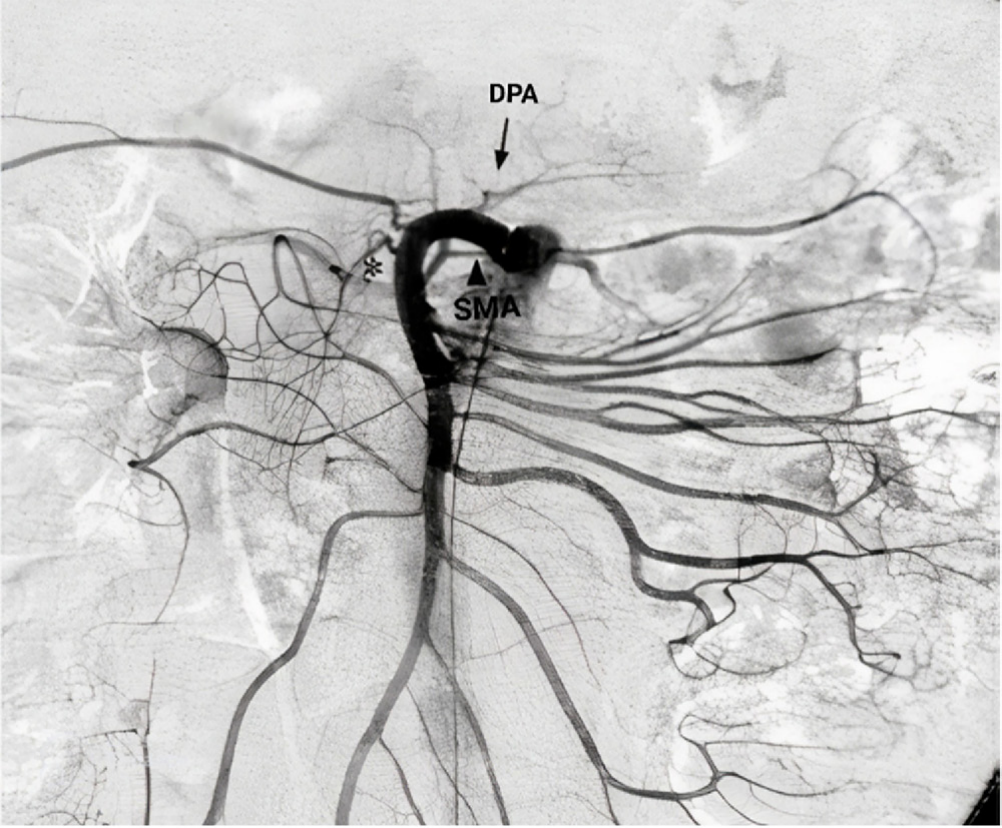
Type II arterial configuration. Note: SMA: superior mesenteric artery, DPA: dorsal pancreatic artery. Source: Okahara et al.^10^

Type II PD arterial configuration, which accounted for 24% of cases, was characterized by the DPA arising from the SMA, which supplies blood to the small intestine, cecum, ascending colon, and transverse colon (Figure 2). The diameter of the DPA was also variable in patients with this variation, ranging from 0.6 mm to 1.8 mm, with an average diameter of 1 mm. The DPA in this configuration coursed posterior to the neck and body of the pancreas, then anastomosed with the inferior PD artery, forming the PD arcade. The inferior PD artery originated from the SMA and coursed anteriorly to the head of the pancreas. This variation was observed in 15 of the healthy participants and 9 participants from the DM/MS group. Some constriction of the pancreatic arteries was observed in a subset of patients. The constriction was primarily observed in Type II arterial variation. In Type II cases, the diameter of the posterior pancreatic artery was usually constricted at the branching point. However, the degree of constriction was not significant enough to be considered pathological or clinically relevant.

**Figure 3:**
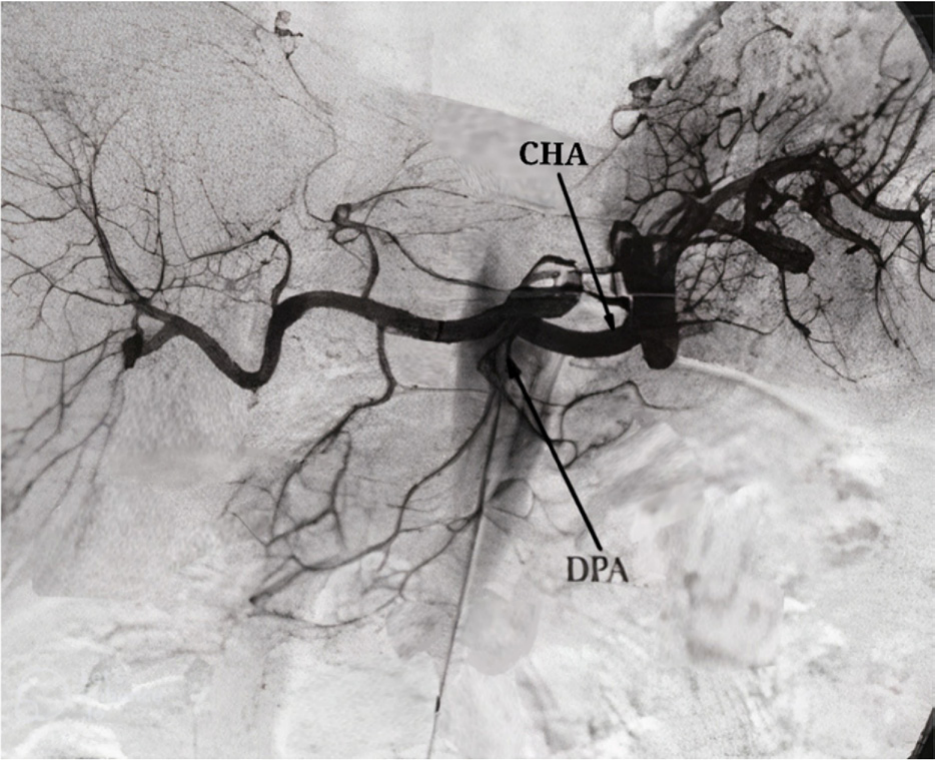
Type III arterial configuration. Note: CHA: common hepatic artery, DPA: dorsal pancreatic artery. Source: Okahara et al.^10^

Type III PD arterial configuration, which was found in only 3% of cases, had unique anatomy compared to the other two types (Figure 3). In this configuration, the DPA arose from the CHA, which is a major branch of the celiac trunk and also supplies blood to the liver, gallbladder, and stomach. The caliber of the posterior pancreatic artery was variable, ranging from 0.8 mm to 1.9 mm, with an average diameter of 1.3 mm. The inferior PD artery, in this case, was a branch of the SMA and coursed anteriorly to the head of the pancreas, where it anastomosed with the DPA to form the PD arcade. This variation was observed in 2 of the healthy participants and 1 participant from the DM/MS group. While this small sample size limits the generalizability of these findings, the patients with Type III arterial variation and diabetes had a longer duration of diabetes compared to the other diabetic patients in the study.

The total case count in the study presents a comprehensive overview of the number of cases for each arterial variation type in the two groups: healthy and DM/MS (Table 1). In this study, the chi-squared tests were used to determine if there was a significant difference in the frequency of each arterial variation type between the two groups.

**Figure 4:**
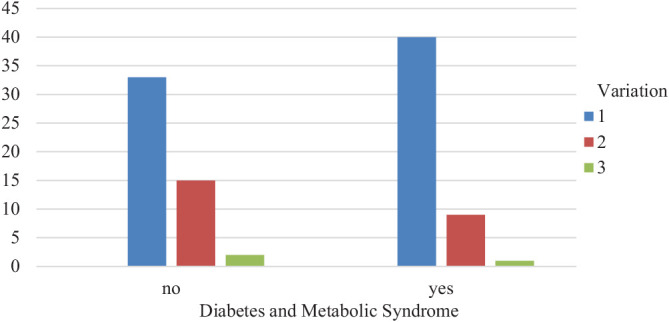
Arterial variation type prevalence in each group. Source: created by the authors.

A *p*-value of less than 0.05 was considered statistically significant. The study results were also presented in a diagram (Figure 4).

Statistical analysis revealed that the distribution of arterial configurations was not significantly different between the healthy group and the group with diabetes and MS (*p* = 0.366) (Table 2). This suggested that the arterial variations the authors observed were not strongly associated with the development of diabetes or MS. It should be noted that the mean age in the group with diabetes and MS was significantly higher compared to the healthy group (*p* < 0.05). The mean age in the diabetes and MS group was 56.5 years, while in the healthy group, it was 47.3 years. On the other hand, there was no significant difference in gender distribution between the two groups (*p* > 0.05). The male-to-female ratio was 1:1.1 in the diabetes and MS group, while it was 1:1 in the healthy group. The study did not consider other factors that could influence the occurrence of DM/MS, such as lifestyle factors, genetic predisposition, and comorbidities. The results of the study suggest that these factors play a more significant role in the development of diabetes and MS than the arterial variations of pancreatic blood flow. Future studies could consider these factors to provide a more comprehensive understanding of the relationship between arterial variations and DM/MS.

The study revealed no significant differences in the prevalence of arterial variations in the blood supply to the pancreas between the groups of patients with diabetes and MS and healthy controls. The analysis showed that the most common configuration of pancreatic arteries is Type I, in which the DPA originates from the SPA. Other variants (Types II and III) were less common. Although some cases of arterial lumen restriction were found, this was not clinically relevant for patients. The identified differences may have clinical implications for planning surgical interventions in patients with pancreatic pathologies, but further studies are needed to assess their impact. An important result is also the confirmation of the effectiveness of MDCT for the study of pancreatic circulation anatomy.

## Discussion

The primary objective of this study was to investigate the correlation between anatomical arterial variations of pancreatic blood flow and the occurrence of DM/MS. Overall, the study found that there were three major different types of arterial configurations in the PD complex and that these configurations were not significantly different between healthy individuals and those with diabetes and MS. However, this study yielded several important findings. Firstly, the study found that MDCT angiography was a useful and reliable imaging modality for studying the vascular anatomy of the pancreas in a safe and comfortable way. MDCT angiography allowed for the identification and classification of three major types of arterial variations in all participants, providing valuable insights into the anatomical variations of the pancreatic blood supply. Secondly, the study identified the prevalence of arterial variations in the study population, which could be extrapolated to the Eastern European population; however, the study was conducted at a single center and was not representative enough. Further studies with larger sample sizes and multicenter designs are needed to acquire more reliable statistics. The three common arterial variations were identified in both patient groups, indicating the importance of recognizing and understanding these variations during clinical and surgical interventions.

The current study explored the potential correlation between arterial variations of pancreatic blood flow and the occurrence of diabetes, a topic of great importance given the significant global burden of this disease. The study identified three major types of arterial variations: Type I, Type II, and Type III. Type III was found to be the rarest variation. In this study, two healthy individuals and one diabetic patient were found to have Type III arterial variation, and it was considered slightly constricted. However, more research was required to establish whether it can significantly affect the blood flow and whether this was a significant finding.

These findings suggest that some degree of arterial constriction could be present in patients with certain variations in the vascular anatomy of the pancreas. However, further research is needed to determine the clinical significance of these findings and their potential impact on surgical planning or interventions for patients with pancreatic diseases or conditions. It should be noted that during the course of this study, MDCT angiography was found to be a highly effective imaging method for assessing the vascular anatomy of the pancreas. It allowed for the identification of arterial variations in all participants and clearly classified these variations. Furthermore, MDCT angiography enabled the researchers to accurately measure the diameter and length of the pancreatic arteries, which was considered important for surgical planning in patients with pancreatic disease. The use of MDCT angiography in this study provided valuable insights into the vascular anatomy of the pancreas and demonstrated its usefulness as a noninvasive imaging method for studying the pancreas and its blood supply. Several studies have also examined the vascular anatomy of the pancreas, such as a study by Appanraj et al.,^4^ in which various imaging techniques were used, such as the “virtual Whipple” technique. In this technique, the arterial phase images were converted into volume-rendered images, where arteries crossing the surgical resection plane were color-coded for easy identification. CT angiography, a diagnostic method similar to the one used in the present study, was also used. The researchers suggested that CT angiography could be a reliable and useful imaging method for assessing the vascular anatomy of the pancreas when used properly. Proper image acquisition and interpretation were stated to be crucial for accurate diagnosis and treatment planning. Their study focused more on the anatomy of the CHA and venous drainage, which also added valuable information on the broader implications of vascular variations in the PD region. Their study utilized more advanced image processing techniques but had a smaller sample than the current study.

The present study has focused primarily on the variations of the DPA and its origins. This approach was chosen because the DPA is one of the most important arterial branches that supply blood to the dorsal portion of the pancreas, and its variations were viewed as the most significant in the development of pancreatic disorders.^9^ The DPA was known to have a complex anatomy and variable origins, which made it difficult to identify and characterize. The anatomy of DPA was also studied by Tatsuoka et al.^11^ in the context of pancreaticoduodenectomy. Their study used thin-slice CT to assess the vascular anatomy of the pancreas, which was rather effective. The results showed that in 68% of the participants, the DPA originated from the celiac axis (CA) or its branches, while in 33% from the SMA or its branches, one patient had two DPAs branching from both CA and SMA. These results differed slightly from the results of the current study—DPA branching from SMA in 24% of cases (Type II variation) and DPA originating from CHA and SPA (branches of the CA) in 76% of participants. The difference could be explained by the different imaging techniques and the small sample size in the current study. Further research is needed to define if the genetic and environmental factors specific to the population could have contributed to these results, as such cases have been described in the literature.^12–14^ Both studies confirmed that arterial variations of the pancreas could have significant implications for patient care and outcomes.

The anatomy of the DPA was comprehensively described by Bertelli et al.^15^ The authors used selective celiac angiography and had a decent sample size (1,015 angiographies). They established that the DPA, which was present in the majority of cases, originated from different arteries such as the SPA, CHA, SMA, or celiac trunk. The findings of the current study were similar to those of previous studies, but the current study benefited from the use of more advanced technology (MDCT), which allowed for higher-quality imaging and more precise data collection. A study conducted by Ibukuro utilized helical CT imaging for purposes similar to the current study.^16^ Helical CT imaging is a powerful tool for visualizing vascular structures, making it possible to identify and characterize arterial variations with high precision and accuracy. Further studies on the topic of arterial variations could benefit from the use of helical CT imaging.

It should be noted that the arterial variations described in the current study are not the only ones that exist. Other anatomical types of arterial blood supply to the pancreas were also described in the literature, such as DPA branching from an accessory middle colic artery,^17^ right gastroepiploic artery,^18^ aberrant common trunk originating from the CHA,^19^ pentafurcated celiac trunk,^20^ and even hexafurcated celiac trunk.^21^ These studies highlighted the complexity of this organ’s vasculature and underscored the importance of further research on this topic. Further studies in this field could also help identify potential risk factors for diseases related to the pancreas and aid in the development of preventive measures and treatments. The correlation between the arterial variations of the pancreas and other diseases was analyzed in the research studies by Rousek et al.^22^ and Schurko et al.^23^ The results were also similar to the current study, despite the different research methods. It was observed that arterial variations in the pancreas primarily had implications for surgical procedures and were not associated with any specific diseases or conditions. While arterial variations did not directly lead to disease development, they were still an important consideration for surgeons performing pancreatic procedures to ensure successful outcomes.^24^

This study has raised several important questions and provided material for further studies. It was observed that the arterial variations did not show any significant difference in terms of the prevalence of DM and MS, but the findings were inconsistent and required further research. Additionally, the findings of this study could be further explored through comparative analyses with other populations, including different age groups, ethnicities, and geographic locations. Finally, using more advanced imaging techniques, such as magnetic resonance imaging, helical CT imaging, or three-dimensional CT (3D CT), could help elucidate the vascular network of the pancreas and aid in the identification of additional arterial variations.

## Conclusion

The present study has provided insights into the arterial variations of the pancreatic region, with a particular focus on the DPA. While the analysis of the correlation between arterial variations and DM/MS did not yield significant results, this study provided a foundation for future research on the topic. The study successfully yielded results on the prevalence of arterial variations in the pancreas. The identification of multiple anatomical types of arterial blood supply suggested that a comprehensive understanding of the pancreatic vasculature is essential for diagnosing and treating pancreatic disorders, especially surgical ones.

The results of this study highlighted the advantages of using MDCT for researching vascular anatomy. This method allowed for the detection of arterial variations that could have been missed with other imaging techniques. The imaging was clear and of high resolution and could be advised for use in future studies regarding this topic. This technique could also be used to investigate the venous and lymphatic drainage of the pancreas, which could play an equally important role. However, it is important to note that MDCT is a relatively expensive imaging modality, and it requires skilled operators to ensure accurate and reliable results.

Future studies on this topic include comparative analyses with other populations (different age groups, ethnicities, and geographic locations), the use of more advanced imaging techniques, and an examination of the potential relationship between arterial variations, venous drainage, measurement of the blood flow in different parts of the pancreas, and other anatomical variations in this organ. Other potential relationships between pancreatic arterial variations and various pancreatic disorders, including chronic pancreatitis, pancreatic cancer, and pancreatic neuroendocrine tumors, could be explored.

## Conflicts of interest

The authors declare that they have no known competing financial interests or personal relationships that could have appeared to influence the work reported in this paper.

## Acknowledgments

N/A.

## Authorship declaration

All authors agree with the content of the manuscript.

## Authors’ contribution

AS: conceptualization, methodology, formal analysis, writing—review and editing; BG: conceptualization, data curation, validation, writing original draft preparation.

## Ethics committee approval

The study was performed per the ethical standards for human research established by the Declaration of Helsinki and Good Clinical Practice guidelines and was approved by the Ethics Committee of the Vasyl Stefanyk Precarpathian National University, IRB number 345-A-001.

## Figures and Tables

**Table 1.** Total case count.

**Count**					
		**Arterial variation type**			**Total**
		**Type I**	**Type II**	**Type III**	
DM and MS	No	33	15	2	50
	Yes	40	9	1	50
Total		73	24	3	100

Note: DM: diabetes mellitus, MS: metabolic syndrome. Source: created by the authors.

**Table 2.** Chi-squared tests.

	**Value**	**Degrees of freedom**	**Asymptotic significance (two-sided)**	**Exact Sig. (two-sided)**
Pearson chi-squared test	2.505	2	0.286	0.366
Likelihood ratio	2.528	2	0.283	0.366
Fisher-Freeman-Halton exact test	2.551	N/A	N/A	0.366
Quantity of valid cases	100	N/A	N/A	N/A

Source: created by the authors.
